# Percutaneous short segmental fixation combined with bone cement augmentation for stage III Kümmell’s disease without nerve deformity

**DOI:** 10.1097/MD.0000000000037087

**Published:** 2024-01-26

**Authors:** Xiang Guo, Yujin Qiu, Xiaowei Liu, Haijun Teng, Hongtao Hu

**Affiliations:** a Department of Orthopedic Surgery, The Affiliated Hospital of Weifang Medical University, Weifang, Shandong, P.R. China.

**Keywords:** augmentation, bone cement, fixation, Kümmell disease, percutaneous, short-segment

## Abstract

The objective of this study was to evaluate the safety and efficacy of percutaneous pedicle screw fixation combined with bone cement augmentation in the management of stage III Kümmell disease without nerve deformity. A retrospective analysis was conducted on 17 patients diagnosed with stage III Kümmell disease without nerve deformity, who underwent treatment with percutaneous pedicle screw fixation combined with bone cement augmentation between April 2019 and 2022. Preoperative, postoperative, and final follow-up clinical outcome measures were collected, including Visual Analog Scale scores and Oswestry Disability Index scores. Additionally, lateral radiography was used to measure the Cobb angle and height of the anterior border of the affected vertebral body. Operative time, volume of injected bone cement, intraoperative cement leakage, and other complications were recorded. All patients underwent successful surgery, resulting in significant reductions in Visual Analog Scale scores, Oswestry Disability Index scores, and Cobb angle postoperatively; meanwhile there was a significant increase in height of the anterior border of the affected vertebral body. No major complications occurred during the follow-up period. In conclusion, percutaneous pedicle screw short-segment fixation combined with bone cement augmentation appears to be an effective surgical option for treating stage III Kümmell disease without nerve deformities.

## 1. Introduction

Kümmell disease (KD) was first reported by Hermann Kümmell in 1895^[1]^ and is characterized by progressive kyphosis and painful vertebral body collapse.^[2]^ Vertebral osteonecrosis, vertebral pseudarthrosis, and intravertebral vacuum sign are terms used to describe the pathological condition of KD.^[3,4]^ Although the pathogenic mechanism of KD is not fully understood, most recent studies indicate that it is associated with nonunion of fractures, impaired osteogenic capability, and local ischemic necrosis of the vertebral body.^[5]^ The most common complications are severe back pain and intravertebral instability, which negatively affect quality of life and do not improve with conservative therapy. Surgical intervention is frequently required for KD.^[6]^ Percutaneous vertebroplasty (PVP) and percutaneous kyphoplasty (PKP) have shown impressive outcomes in patients with stages I and II KD.^[7–10]^ However, this is inappropriate for stage III KD, which is accompanied by kyphosis, intravertebral instability, or nerve deformity. In the past, operations such as posterior decompression with pedicle subtraction osteotomy, anterior decompression, and reconstruction, or a combined anterior and posterior approach, were used to treat these cases and achieved good results.^[11–15]^ Due to their time-consuming procedures, potential for substantial trauma, bleeding, and other consequences, or the common use of bone graft fusion with internal fixation of a pedicle screw, they are contraindicated in elderly patients with significant comorbidities. Recently, short-segment pedicle screw fixation with PVP has been found to be more effective in the treatment of stage III KD with neurological deficits.^[16,17]^ However, decompression and open surgery are not required for stage III KD without neurological deficits. Therefore, we hypothesized that percutaneous pedicle screw short-segment fixation combined with vertebroplasty could be an effective and safe procedure in patients with stage III KD without neurological deficits. In this study, 17 patients underwent percutaneous pedicle screw short-segment fixation combined with vertebroplasty. Here, we report the details of the operation and related complications and summarize the clinical efficacy and radiological follow-up.

## 2. Materials and methods

### 2.1. General information

In this study, we collected the data of 17 patients with typical clinical symptoms and complete imaging data at the Department of Orthopedic Surgery, Affiliated Hospital of Weifang Medical College, from December 2019 to April 2022. There were 6 males and 11 females aged 67 to 85 years (average 75.2 ± 5.7 years). The preoperative, bone mineral density T-score measured via dual-energy X-ray absorption ranged from –2.6 to –4.3 (mean –3.3 ± 0.4). The fracture sites were: T_11_ 2, T_12_ 3, L_1_ 8, and L_2_ 4. The clinical manifestation was intractable pain in the lower back, which was aggravated especially during postural changes, and the pain range was consistent with the range of the injured vertebrae. Preoperative imaging revealed typical intravertebral “vacuum sign” changes and posterior convexity deformities of the affected segments to varying degrees. General patient information is shown in Table 1.

**Table 1:** Summary of data obtained from 17 patients with Kümmell disease.

Case no	Age/sex	BMD	Trauma history	Diseased level	Follow-up months	Operation time (min)	Cement volume (mL)	Intraoperative blood loss (mL)
1	68/M	−2.6	Flat falling	L2	12	90	12	35
2	76/M	−3.1	Flat falling	L1	14	76	12	40
3	80/F	−3.3	Flat falling	L1	13	73	13	41
4	83/F	−2.8	Lumbar strain	L2	13	80	12	34
5	72/F	−3.4	Flat falling	L1	10	76	9.5	44
6	76/F	−3.1	No	L1	6	72	10	33
7	76/F	−3.1	Flat falling	L1	11	62	11	48
8	81/F	−3.5	Flat falling	T12	12	65	10	32
9	85/F	−3.3	No	L1	12	66	10.5	46
10	76/M	−3.7	Flat falling	T11	8	80	9	45
11	71/F	−3.2	Lumbar strain	L2	14	82	11	38
12	77/M	−3.4	No	L2	9	76	8.5	46
13	75/M	−2.9	No	T12	11	66	6.5	35
14	81/M	−4.3	Flat falling	T11	13	84	12	45
15	67/F	−3.1	Flat falling	T12	10	75	9	38
16	68/F	−3.3	Lumbar strain	L1	15	95	8	41
17	67/F	−3.2	Flat falling	L1	12	88	10	36

BMD = bone mineral density, F = female, M = male.

This study was approved by the Institutional Review Board (IRB) of the Affiliated Hospital of Weifang Medical College (IRB number: wyfy-2021-ky-125) and was performed in accordance with the Declaration of Helsinki. The requirement for informed consent (written/verbal) of the patients was waived because of the retrospective nature of this study.

### 2.2. Inclusion and exclusion criteria

The inclusion criteria were as follows: stage III KD in which single-level vertebral body delayed osteonecrosis with an intravertebral vacuum cleft and spine kyphosis deformity; minor trauma (fall or strain) followed by pain that worsens with postural changes, lasts for at least 4 weeks on average, and leads to kyphosis; magnetic resonance imaging, CT, and X-ray scan showing typical “vertebral body vacuum fissure” sign or effusion, with the posterior wall of the body intact^[18]^; age > 55 years and a bone mineral density T value ≤ –2.5; and no obvious nerve or spinal cord compression or injury.

The exclusion criteria were as follows: symptoms of nerve injury; malignant tumor, infection, metabolic bone disease, or coagulation dysfunction; and severe compression of the vertebral body, rendering puncture impossible.

### 2.3. Surgical procedure

All the surgical procedures were performed by an experienced spinal surgeon. The patients were placed in the prone position under general anesthesia with endotracheal intubation. The chest and iliac regions were elevated using soft cushions, and the surgical bed was adjusted such that the patient was in a hyperextended prone position. Using a C-arm X-ray fluoroscope, the projected positions of the pedicle of the injured vertebra and the adjacent upper/lower segments were marked on the body surface preoperatively.

After disinfection and placement of sterile drapes, 4 hollow pedicle screws were placed percutaneously in each of the adjacent upper and lower vertebrae of the injured vertebra under C-arm X-ray monitoring. If the vertebral body was severely osteoporotic, a small amount of bone cement was injected into the vertebral body before the pedicle screw was placed. Re-bent longitudinal connecting rods were inserted into the U-shaped slots of screws under the paraspinal muscles. The vertebral body was braced and repositioned via an extracorporeal bracing system, and the height of the diseased vertebral body was significantly elevated on fluoroscopy compared with the preoperative height. Subsequently, a trocar of 5 mm diameter was inserted into the injured vertebral body and placed in the anterior 1/3 of the vertebral body. Bone cement was pushed into the injured vertebra after preparation between the medial wall of the arch and the median line using a fractional infusion technique. Fluoroscopy revealed that the bone cement was well-filled. The wound was closed layer-by-layer and covered with sterile excipients. Drainage tubes were not required.

The minimally invasive internal fixation stabilization system was supplied by Shandong Weigao Orthopedic Instruments Company of China, the VP system was supplied by Shandong Guanlong Company of China, and polymethylmethacrylate bone cement was supplied by Tecres S.P.A. Tecres S.p.A., Italy.

### 2.4. Postoperative management

**Figure 1.**
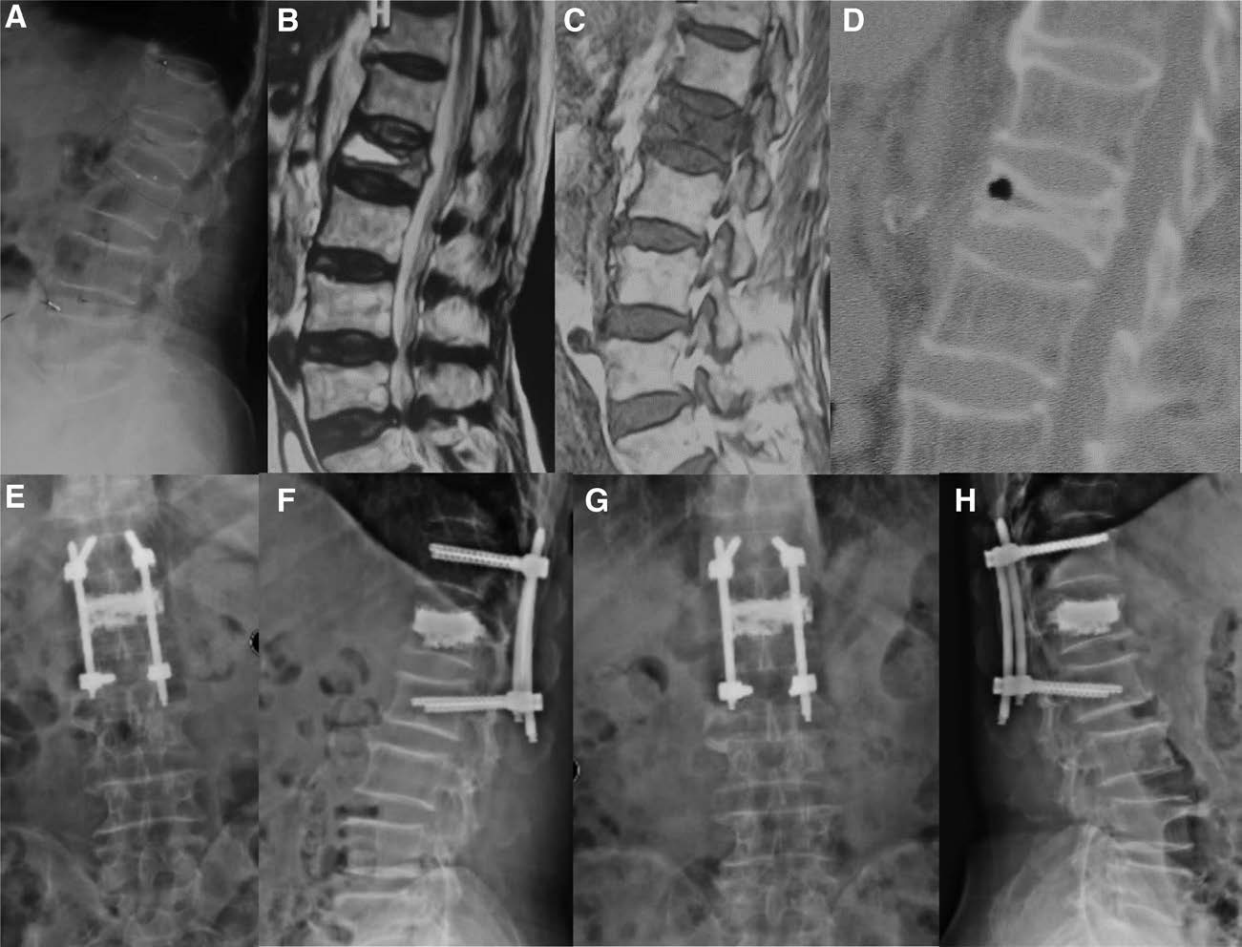
A 76-year-old-male patient who underwent percutaneous fixation combined with vertebroplasty for L1 Kümmell disease. (A–D) Preoperative imaging showed L1 vertebral fracture with “vertebral body vacuum fissure” sign. (E and F) Postoperative X-ray showed fine bone cement position and injury vertebral body height recovery. (G and H) Postoperatively 12 months X-ray showed firm internal fixation, stable vertebral body height, and fine bone cement position.

Postoperative antibiotics were routinely administered to prevent infection and discontinued 24h after surgery. All patients were encouraged to stand and walk on the 1^st^ day postoperative day. Bisphosphonates, vitamin D, and calcium supplements were routinely administered to each patient to treat osteoporosis. The brace was worn for 4 to 6 weeks after surgery, and functional exercise of the lumbar back muscles was initiated in the first month after surgery. All patients were examined preoperatively, at 1, 3, 6, and 12 months postoperatively, and annually thereafter. A typical case is shown in Figure 1.

### 2.5. Observational indicators

Operating time and intraoperative bleeding of the patients were recorded. The level of pain was assessed using a visual analog scale (VAS) ranging from 0 to 10, with 10 indicating the most severe pain. The level of disability was evaluated using the Oswestry Disability Index (ODI) of 100, with a higher score indicating more severe pain and disability. Preoperative imaging was performed using standing anteroposterior and lateral radiographs, CT scans, and MR imaging. The height of the anterior border of the injured vertebral body (HAV) and Cobb angle (CA) were determined using Cobb method of measurement between the superior endplate plumb line of the superior vertebral body and the inferior endplate plumb line of the inferior vertebral body were analyzed using serial radiographs. The loss of correction was calculated using the difference between the postoperative and final follow-up. All imaging measurements were analyzed using the image archiving and transmission system (PACS) and its accompanying software system.

### 2.6. Statistical analysis

Data entry and analysis were performed using SPSS 23.0 statistical software (IBM SPSS Corp. Armonk, NY). The data were expressed as the mean ± standard deviation. The Student *t* test was used to evaluate VAS, ODI, CA, and HAV changes based on the data obtained preoperatively, postoperatively, and at the final follow-up. The level of statistical significance was set a *P* < .05.

## 3. Results

All patients successfully completed the surgery. The mean operative time was (76.8 ± 9.2) min, the bleeding volume was (39.8 ± 5.1) mL, and the amount of bone cement injected was approximately (10.2 ± 1.6) mL. In one case, the screw was pulled outward during propping and bone cement was used to reinforce it. No leakage of bone cement from the venous plexus or spinal canal occurred in any case. None of the patients experienced any postoperative complications such spinal nerve damage.

**Figure 2.**
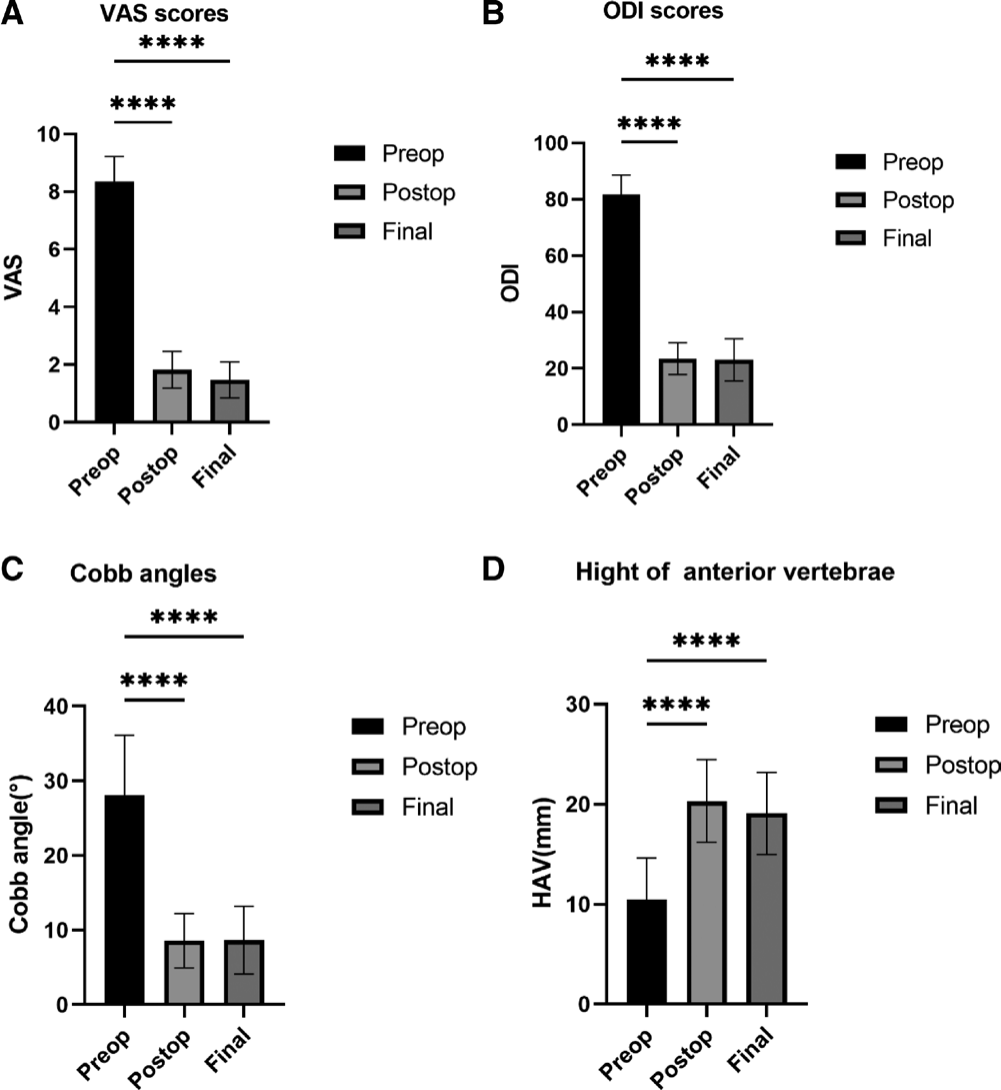
The VAS and ODI scores, the Cobb angles and the height of the anterior border of the injured vertebral body. (A) The VAS scores at preoperation; and postoperation and the final follow-up after surgery were significantly better than those before surgery. (B) The ODI scores at pre-operation; and post-operation and the final follow-up after surgery were significantly better than those before surgery. *P < .01 compared to the preoperative scores. The Cobb angles and the height of the anterior border of the injured vertebral body. (C) The Cobb angles at pre-operation; and post-operation and the final follow-up after surgery were significantly better than those before surgery. (D) The height of the anterior border of the injured vertebral body at pre-operation; and post-operation and the final follow-up after surgery were significantly better than those before surgery. *P < .01 compared to the preoperative scores. ODI = Oswestry Disability Index, VAS = visual analogue scale.

The mean VAS score and ODI significantly decreased from 8.3 ± 0.8 and 81.6% ± 6.9% preoperatively to 1.8 ± 0.6 and 23.4% ± 5.6% postoperatively, respectively (*P* < .01). At the final follow-up, the mean VAS score and ODI were 1.4 ± 0.6 and 23.0% ± 7.4%, respectively, which were well maintained (*P* > .05) (Fig. 2A and B). The postoperative follow-up radiograph showed that the pedicle screws were well positioned, no poorly positioned or extracted pedicle nails were found, and the injured spine was well filled with bone cement, with no leakage in the spinal canal. No loosening or displacement of the cement in the injured spine or loosening and fracture of the screws were found at the time of follow-up. There was a significant correction of CA and HAV from 28.1° ± 7.9° and 10.1 mm ± 4.1 mm preoperatively to 8.5° ± 3.6° and 20.1 mm ± 4.1 mm postoperatively, respectively (*P* < .01). There was no significant loss in the local CA and HAV at the final follow-up (CA, 8.6° ± 4.5°; HAV, 18.7° ± 4.1°) (*P* > .05) (Fig. 2C and D).

## 4. Discussion

With the increasing aging of society and the continuous progress of imaging technology, especially magnetic resonance imaging, the literature on KD has shown an increasing trend.^[11]^ Recently, surgical intervention has emerged as the preferred treatment option. A minor procedure known as PVP or PKP has been shown to stabilize the vertebral body and relieve back pain.^[19–22]^ However, its effectiveness in correcting kyphosis is limited, and tends to result in bone leakage.^[23,24]^ Therefore, PVP or PKP are mostly used in KD at stages I and II. As the disease progresses to stage III, it will gradually present vertebral collapse, kyphosis, intravertebral instability, and spinal nerve compression occur. Several surgical techniques, including fixation combined with vertebroplasty, open reduction, posterior osteotomy, and anterior vertebrectomy, have been used to treat stage III KD.^[25–31]^ Based on Li staging, Wang further divided stage III KD into 3 subtypes according to the presence of preoperative neurological symptoms and source of neurological compression in patients.^[32]^ Spinal canal decompression is required for KD at stage IIIc, which results in substantial nerve compression. However, for patients with stage IIIa or IIIb KD, who have no neurological symptoms or mild neurological symptoms, the focus of treatment is the correction of kyphosis and restoration of spinal stability. The use of bone-filling mesh containers can partially restore vertebral body height; however, bracing strength is frequently insufficient to improve the rate of deformity correction.^[33]^ Additionally, balloon expansion followed by bone cement injection may result in postoperative cement loosening owing to insufficient adhesion of the cement to the surrounding bone tissue.^[24]^ is A common treatment for kyphosis or spinal fractures without neurological symptoms.^[12,16,17,34]^ However, patients with KD have less bone mass, and there is a risk of screw loosening and removal over time. Therefore, some studies have claimed in certain research that the use of bone cement to reinforce the nail path can increase the strength of the control force based on open reduction and internal fixation. As most patients with KD are in poor physical condition, have severe osteoporosis, and have additional underlying medical conditions, it is important to use trauma-minimizing strategies to relieve symptoms and restore lumbar stability. While less traumatic and requires less time in surgery than open screw fixation, percutaneous screw fixation has equivalent efficacy. These factors are crucial for patients with KD. Using a short-segment pedicle screw, which not only produces a stable reduction effect on the anterior and middle columns, but can also restore the fracture block to its original position by using the tension of the posterior longitudinal ligament and indirectly expanding the space of the spinal canal, it is direct and reliable to restore the height of the injured vertebra.^[35–37]^

All patients included in this study had KD at stages IIIa or IIIb. Decompression was not the main objective of the procedure, because the nerves in both subtypes were either uncompressed or moderately compressed. Therefore, kyphosis can be treated and spinal stability can be restored using a minimally invasive percutaneous procedure. After assuming a hyperextended prone position under general anesthesia during the preoperative period, the posterior convexity can be somewhat improved. The injured spine can then be completely repositioned using percutaneous pedicle screw short-segment fixation and extracorporeal bracing. Clinical observations revealed that it reduced the CA and increased the height of the anterior borders of injured vertebrae. The fractured vertebra is then propped open, creating a negative chamber that resembles an eggshell and lowers the pressure of the bone-cement injection and the risk of leaking. Additionally, it encourages efficient dispersion of bone cement into the loose bone trabecular area, fills the gap between injury and necrosis, and more effectively rebuilds the anterior and middle columns of the injured vertebra. Spinal stability was restored by injecting bone cement into the injured spine via a percutaneous vertebroplasty system, which allowed the necrotic area of the injured spine to be filled. The VAS and ODI scores were significantly lower. Furthermore, posterior percutaneous pedicle nailing can completely correct kyphosis and restore the sagittal spinal balance.^[38]^ In this study, we used adjacent vertebral body internal fixation to effectively restore vertebral body height and distribute local vertebral body stress. No complications such as vertebral body refracture or cement loosening and displacement were observed during the postoperative follow-up. To avoid screw pullout during bracing in some patients with severe osteoporosis, we used cemented nail tract strengthening to improve the screw pullout resistance. During the follow-up period, no signs of internal fixation failure such as screw loosening or pullout were observed. None of the patients underwent neurological decompression because of mild neurological symptoms. However, the patient neurological function improved to some extent after surgery, which could be attributed to factors such as reduced neural provocation after postoperative spinal stability reconstruction, and reduced neural compression after correction of the retroflection deformity.^[37]^

The advantages of minimally invasive percutaneous surgery over traditional open surgery^[39]^: less trauma, less time, and less bleeding, requiring less surgical tolerance; less damage to the muscles and soft tissues surrounding the spine during surgery, which is beneficial to postoperative recovery; and percutaneous pedicle nailing at the upper and lower ends of the injured spine can disperse postoperative stress on the injured spine and reduce distant cement loosening, displacement, and fracture risk. To avoid internal fixation failure in such patients, the authors recommend effective postoperative antiosteoporotic therapy. If the restoration is unsatisfactory, special bracing instruments can be used after intraoperative pedicle nail insertion, and bilateral bracing must be performed to avoid pedicle nail extraction due to excessive bracing force.^[28]^ Because there are frequent fissures in the 4 walls of the injured vertebral body, there is a risk of cement leakage during vertebral body strengthening; therefore, close fluoroscopic observation of cement dispersion is required during cement injection.^[40]^

The limitations of this study are the lack of comparison groups of patients treated with either conservative treatment or VP alone, as well as its relatively small sample size due to the rarity of KD. Furthermore, the precise circumstances surrounding the long-term complications were unclear because of the relatively short follow-up period. Therefore, more prospective and long-term follow-up studies are needed to elucidate this issue further.

## 5. Conclusion

In conclusion, percutaneous pedicle screw short-segment fixation with bone cement augmentation appears to be an effective surgical option for treating stage III KD without nerve deformities. It can correct kyphosis, restore spinal stability, and relieve pain quickly. Clinical efficacy was achieved with a short operation time, little trauma, less bleeding, fewer surgical complications, and a short postoperative bed rest time, which is especially important for elderly patients in a poor general condition.

## Acknowledgments

The authors would like to express their gratitude to Preflight (https://preflight.paperpal.com/) for the expert linguistic services provided.

## Author contributions

**Conceptualization:** Yujin Qiu, Haijun Teng, Hongtao Hu.

**Data curation:** Xiang Guo, Xiaowei Liu.

**Formal analysis:** Xiang Guo.

**Investigation:** Xiaowei Liu.

**Methodology:** Xiang Guo, Yujin Qiu, Hongtao Hu.

**Project administration:** Hongtao Hu.

**Writing – original draft:** Xiang Guo, Hongtao Hu.

**Writing – review & editing:** Hongtao Hu.
